# Surface Nanostructuring during Selective Area Epitaxy of Heterostructures with InGaAs QWs in the Ultra-Wide Windows

**DOI:** 10.3390/nano11010011

**Published:** 2020-12-23

**Authors:** Viktor Shamakhov, Dmitriy Nikolaev, Sergey Slipchenko, Evgenii Fomin, Alexander Smirnov, Ilya Eliseyev, Nikita Pikhtin, Peter Kop`ev

**Affiliations:** 1 Ioffe Institute, 26 Politekhnicheskaya, St Petersburg 194021, Russia; dim@mail.ioffe.ru (D.N.); serghpl@mail.ioffe.ru (S.S.); alex.smirnov@mail.ioffe.ru (A.S.); ilya.eliseyev@mail.ioffe.ru (I.E.); nike@mail.ioffe.ru (N.P.); Ps@kopjev.ioffe.ru (P.K.); 2 Elfolum Ltd., 26 Politekhnicheskaya, St Petersburg 194021, Russia; evgeny.fomin@bk.ru

**Keywords:** MOCVD, selective area epitaxy, selective area growth, photoluminescence, atomic force microscopy, semiconductors, quantum wells, InGaAs

## Abstract

Selective area epitaxy (SAE) is widely used in photonic integrated circuits, but there is little information on the use of this technique for the growth of heterostructures in ultra-wide windows. Samples of heterostructures with InGaAs quantum wells (QWs) on GaAs (100) substrates with a pattern of alternating stripes (100-μm-wide SiO_2_ mask/100-μm-wide window) were grown using metalorganic chemical vapour deposition (MOCVD). It was found that due to a local change in the growth rate of InGaAs QW in the window, the photoluminescence (PL) spectra measured from the edge to the center of the window exhibited maximum blueshifts of 14 and 19 meV at temperatures of 80 K and 300 K, respectively. Using atomic force microscopy, we have demonstrated that the surface morphologies of structures grown using standard epitaxy or SAE under identical MOCVD growth conditions correspond to a step flow growth with a step height of ~1.5 ML or a step bunching growth mode, respectively. In the structures grown with the use of SAE, a strong variation in the surface morphology in an ultra-wide window from its center to the edge was revealed, which is explained by a change in the local misorientation of the layer due to a local change in the growth rate over the width of the window.

## 1. Introduction

The monolithic integration of electro-optical elements has recently been the subject of extensive research and development in the field of photonic integrated circuits [1,2,3,4,5,6] with various functions including control and generation of both optical (multi-wavelength laser sources, modulators, low-loss waveguides, splitters, combiners, etc.) and electrical signals. Selective area epitaxy (SAE) [7,8,9] is one of the effective approaches to the implementation of these elements, using selective growth on patterned substrates with passivating masks, which exclude growth above them and ensure growth outside (i.e., in the so-called windows). There are a number of works demonstrating the use of SAE in the fabrication of different optical devices based on various material systems, including III-V (GaAs [8,10,11], InP [12,13,14,15,16,17]) and III-N semiconductor compounds (GaN [18], InGaN [19]). In particular, single-mode lasers with monolithically integrated modulators [20,21] and couplers [22,23] have been demonstrated. The applications of SAE for forming nano-objects, including quantum dots [24,25,26,27] and nanowires [28,29], are actively being developed.

Using SAE, one can significantly modify the active region of semiconductor lasers and, thus, fabricate multi-wavelength single-mode laser arrays [17,30], monolithic semiconductor sources of fs laser pulses [31], and tunable semiconductor lasers with an ultra-wide tuning range [15]. Moreover, the ability of SAE to realize new designs of high-power semiconductor lasers that require the formation of lateral waveguide and a spatially configured active region [32] should be emphasized.

One of the important features of SAE is the dependence of the composition and properties of epitaxial layers grown using this method on the geometric dimensions of the masked regions and windows [33,34]. This dependence is caused by the mass conservation during growth that leads, on the one hand, to a decrease in the growth area due to the inhibition of growth in the masked regions. On the other hand, this results in growth rate enhancement (GRE) in the windows. Previously developed models suggested that the changes in the growth rate induced by the mask are determined by the composition of the deposited mask material and various limiting factors, such as diffusions at the surface [35] and/or in the vapor phase [36,37]. Comprehensive theoretical analysis of these phenomena becomes more complicated when simulating ternary and quaternary alloys compared to binary ones. It should be noted that precise knowledge of the MOCVD growth conditions determined by numerous parameters such as pressure distribution in the reactor, the growth rate, and the substrate temperature, etc. is important for the successful simulation.

Although there are quite a few articles on the SAE growth features and SAE-based devices, the published studies are limited to either demonstrating epitaxial layer profiles reflecting the GRE distribution in the window [10,37,38], or demonstrating the overall radiative characteristics of devices [10,20,37]. Although spatial distribution of the composition can be simulated using the experimental spatial distribution of the GRE [20,37], such simulations are not accurate enough. This is due to the fact that the models have adjustable parameters (for example D/k), which are determined as a result of the best match between the measured and calculated GRE profiles. In addition, a key feature of SAE is the spatial inhomogeneity of the thickness and composition of the layers grown in the window, which determines the optical characteristics of SAE-based optoelectronic devices and photonic integrated circuits. Currently, there are no experimental studies showing the composition distribution in the window, as well as SAE growth modes. Moreover, most articles on SAE describe the growth in relatively narrow windows with a width ranging from a few to tens of microns [39]. Thus, the available theoretical and experimental background is insufficient for the development of large planar structures suitable for the fabrication of various types of light-emitting devices, such as semiconductor lasers and multi-wavelength laser arrays.

In this paper, for the first time, the features of the SAE growth of both GaAs bulk layers and InGaAs/GaAs quantum wells (QWs) were experimentally studied using microphotoluminescence, atomic force microscopy, and profilometry. We have demonstrated and discussed spatial distribution of optical characteristics and growth modes of these layers and structures in ultra-wide windows.

## 2. Materials and Methods

### 2.1. Samples

The samples were grown on n-GaAs(100) substrates using MOCVD setup EMCORE GS3100 with a vertical reactor and resistive heating of the substrates. The substrates were misoriented up to 0.2°. Trimethylgallium (TMGa), trimethylaluminum (TMAl), and trimethylindium (TMIn) (Elma-Chem, Zelenograd, Russia) were used as sources of group III atoms, while arsine and hydrogen were used as the source of group V atoms and carrier gas, respectively. The reactor pressure was maintained at 77 Torr. The substrate temperature and rotation speed were 700 °C and 1000 rpm, respectively.

**Figure 1:**
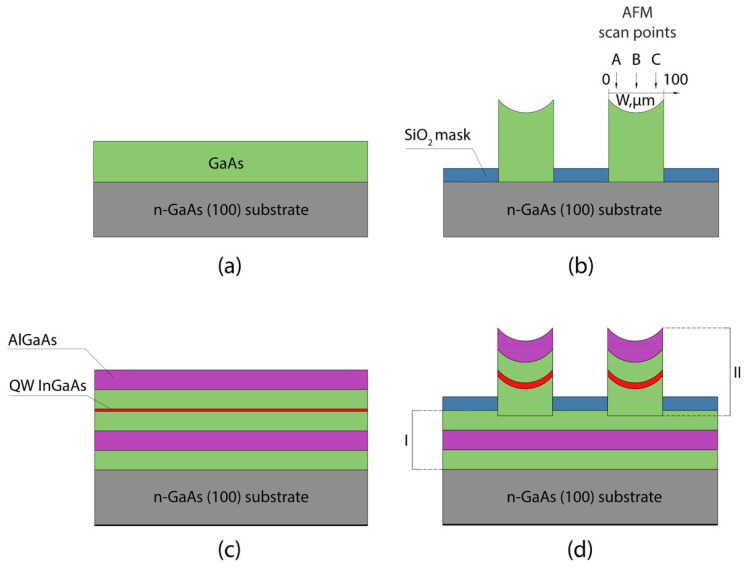
Schematic views of samples grown using (**a**) standard MOCVD of GaAs layers (St-L-L and St-L-H) and (**b**) Selective area epitaxy (SAE) of GaAs layer on the patterned substrate with window/mask widths of 100/100 µm (SAE-L-W) (atomic force microscope (AFM) scan points: A—12–17 µm, B—48–53 µm, C—85–90 µm), as well as samples of heterostructures with InGaAs quantum wells (QWs) grown using (**c**) standard MOCVD (St-ML) and (**d**) SAE on the patterned substrate (SAE-ML-W) (I—standard epitaxy, II—SAE). See description of samples in Table 1.

Figure 1 schematically shows the samples under study. Figure 1a,c show samples obtained with standard MOCVD on a conventional substrate. Figure 1a shows a sample with a standard GaAs layer (St-L). Two 0.18 µm-thick GaAs samples were grown at the rates of 18.8 nm/min (St-L-L) and 37.6 nm/min (St-L-H), respectively. Figure 1c shows the standard-grown heterostructure/multilayer structure (St-ML) with a 0.3-μm-thick GaAs buffer layer, a 0.4-μm-thick Al_0.3_Ga_0.7_As lower cladding layer, a 0.21-μm-thick GaAs lower waveguide layer, a 4.3-nm-thick InGaAs QW, a 0.2-μm-thick GaAs upper waveguide layer, and a 0.1-μm-thick Al_0.3_Ga_0.7_As upper cladding layer.

SAE samples with a GaAs layer and an InGaAs/GaAs heterostructure had 50-nm-thick SiO_2_ dielectric masks deposited using reactive ion–plasma sputtering on the substrate and GaAs intermediate layers as shown in Figure 1b,d, respectively. Then, two types of mask patterns with alternating mask/window stripes of different widths were formed using wet chemical etching. Samples denoted by letters N and W had window/mask stripe widths of 60/340 µm and 100/100 µm, respectively. All St-L and SAE-ML samples were grown under the same MOCVD growth conditions, respectively. SAE-ML samples were grown using two steps. Initially, a 0.3-μm-thick GaAs buffer layer, a 0.4-μm-thick Al_0.3_Ga_0.7_As lower cladding layer, and a 0.3-μm-thick GaAs lower waveguide layer were grown on a bare substrate. Then, a SiO_2_ mask was deposited and etched to form a W pattern. It should be noted that GaAs layers had an etch depth of 0.1 µm in the windows to eliminate possible contamination defects. Taking into account GRE for the W pattern, the lower waveguide layer with a thickness of 0.09 µm in the center of the window was grown. Then, an InGaAs QW was grown using the same growth parameters (QW growth time) as for the growth of the St-ML sample. Finally, an upper waveguide GaAs layer 0.2 µm thick and an upper cladding Al_0.3_Ga_0.7_As layer 0.1 μm thick were grown. Samples parameters are given in Table 1.

The surface morphologies of the samples were studied with an NTEGRA atomic force microscope (AFM) (NT-MDT Spectrum Instruments, Zelenograd, Russia) using transverse scanning in a semi-contact mode at frequency of 1 Hz, an image resolution of 400 points, ETALON HA_FM probes with a resonance frequency of 114 kHz. In addition, to estimate GRE in the ultra-wide windows, the profile of GaAs layers after removing the SiO_2_ mask was measured using a surface profilometer AmBios XP-1.

The micro-photoluminescence (µ-PL) measurements were performed both at room and at 80 K temperatures using a T64000 (Horiba Jobin Yvon, Kyoto, Japan) spectrometer equipped with a confocal microscope. These spectra were measured using the continuous-wave (cw) excitation at 532 nm (2.33 eV) of a Nd:YAG laser (Torus, Laser Quantum, Stockport, UK) with a power on the samples as low as ~40 µW. The spectra were recorded using a 600 lines/mm grating and liquid-nitrogen-cooled charge-coupled device (CCD) camera with Mitutoyo 100 × NIR (NA = 0.90) long working-distance objective lens to focus the incident beam into a spot of ~2 μm diameter. The measurements were carried out with point-to-point scanning with a step of 5 μm. The µ-PL spectra from St-ML and SAE-ML samples were measured at 80 K and 300 K. In addition, µ-PL spectra were measured for AlGaAs upper cladding layer at 80 K. A Linkam THMS600 (Tadworth, UK) temperature-controlled microscope was used for the low-temperature µ-PL measurements.

### 2.2. SAE Simulation Model

**Figure 2:**
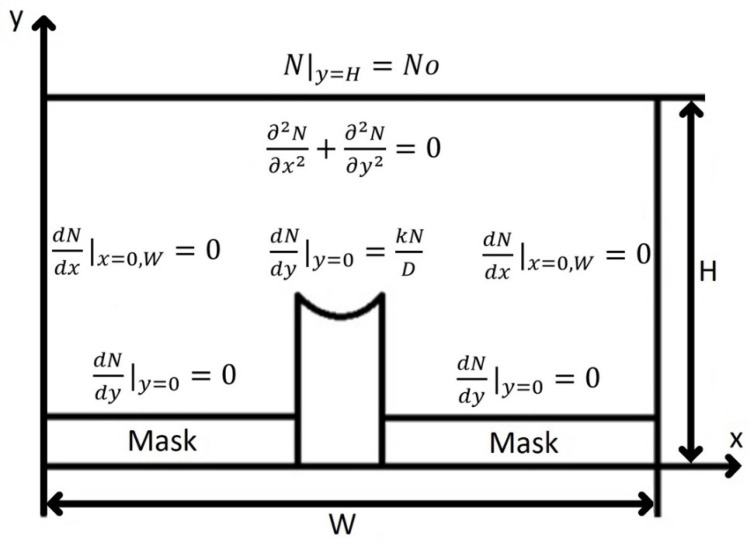
Cross-section of the vapor-phase diffusion simulation domain on a substrate with a window in an oxide mask, where *N* is a precursor concentration, *N_0_* is a precursor concentration at the top of the boundary layer, *W* and *H* are the width and the thickness of the boundary layer, *D* is the mass diffusivity constant, and *k* is the surface reaction constant.

We used the vapor-phase diffusion model [9] for SAE simulations. The model is based on calculating the concentration profile of particles in the gas phase over the substrate surface. The profile is obtained by solving the Laplace equation in the boundary layer domain of width *W* and height *H*. Figure 2 shows the SAE diffusion model.

The boundary conditions at the edges of the boundary layer domain can be written as:

(1) $$N{|}_{y=H}=No,$$

(2) $$\frac{dN}{dx}{|}_{x=0,W}=0,$$

(3) $$\frac{dN}{dy}{|}_{y=0}=0,$$

(4) $$\frac{dN}{dy}{|}_{y=0}=\frac{kN}{D}.$$

Boundary condition (1) implies that the upper part of the boundary layer should be located at a sufficiently large distance from the substrate to avoid any fluctuations introduced by the mask. Boundary condition (2) implies that the precursor concentration within the boundary layer does not vary laterally. Boundary condition (3) shows that the precursor is not deposited on the surface when it reaches the mask surface and follows from Fick′s law. Boundary condition (4) shows that the precursor is deposited on the surface when it reaches the semiconductor surface, and is derived from Fick′s law and the Langmuir isotherm.

The precursor concentration profile is determined by the ratio *D/k*, which can be considered as the effective diffusion length. *D/k* can be estimated either by theoretical calculation or by fitting to experimental data.

The diffusion coefficient *D_AB_* can be calculated theoretically by solving the Boltzmann Equation:

(5) $$D_{AB}=\frac{0.00266T^{3/2}}{PM_{AB}^{1/2}\sigma_{AB}^{2}{Ω}_{D}},$$

(6) $$\sigma_{AB}=\frac{1}{2}\left(\sigma_{A}+\sigma_{B}\right),$$

(7) $${Ω}_{D}=\frac{1.06036}{{\left(T^{*}\right)}^{0.1561}}+\frac{0.193}{e^{\left(0.47635T^{*}\right)}}+\frac{1.03587}{e^{\left(1.52996T^{*}\right)}}+\frac{1.76474}{e^{\left(1.76474T^{*}\right)}},$$

(8) $$T^{*}=\frac{k_{b}T}{\varepsilon_{AB}},$$

(9) $$M_{AB}=2\frac{M_{A}M_{B}}{M_{A}+M_{B}},$$

where *T* is the growth temperature; *P* is the reactor pressure; *M_AB_* is the reduced molecular weights of TMGa and H_2_ (reduced molecular weights of gases A and B); *M_A_* and *M_B_* are molecular weights of TMGa and H_2_, respectively; *k_b_* is the Boltzmann constant; *Ω_D_* is the diffusion collision integral; *σ_AB_* is the characteristic length of the intermolecular force law; *σ_A_* and *σ_B_* are characteristic values of the intermolecular forces between TMGa and H_2_ molecules, respectively; *ε_AB_* is the energy of the Lennard-Jones theorem; *ε_A_* and *ε_B_* are energy parts of Lennard-Jones coefficient for TMGa and H_2_, respectively; T* is a function of the Lennard-Jones parameters.

The following values of the Lennard-Jones parameters were used for TMGa and H_2_: *M_TMGa_* = 114.83 g/mol, *ε_TMGa_/k_b_* = 398 K, *σ_TMGa_* = 5.68 Å, *M_H2_*= 2.016 g/mol, *ε_H2_/kb* = 59.7 K, *σ_H2_* = 2.827 Å.

The rate constant of the surface reaction *k* is calculated by:

(10) $$k=\frac{1}{4}\left(\frac{\gamma }{1-\frac{\gamma }{2}}\right)\sqrt{\frac{8k_{b}T}{\pi M}},$$

where γ is the sticking coefficient.

The GRE concept is used to estimate the profile of the layer grown in the mask window. GRE characterizes the change in the growth rate of a SAE grown layer as compared to a standard growth. GRE is calculated by:

(11) $$GRE=\frac{N}{N_{0}}\left(1+\frac{H}{\frac{D}{k}}\right).$$

## 3. Results and Discussion

### 3.1. Growth Rate Enhancement (GRE) Effect in GaAs Layers Grown by SAE

**Figure 3:**
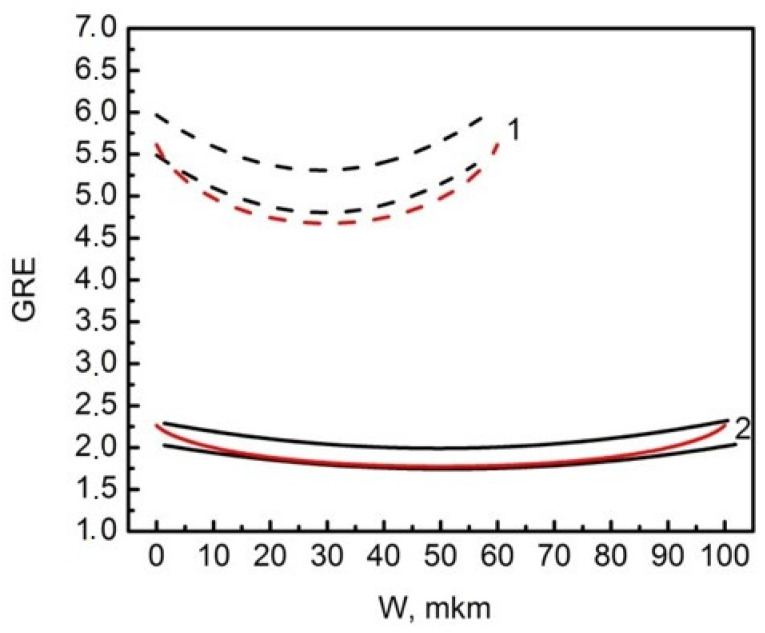
GRE profiles across the window for GaAs SAE-L-N (1) and SAE-L-W (2) samples grown by the SAE technique at the same growth reactor parameters corresponding to a growth rate of 18.8 nm/min for the St-L-L sample.

The GRE effect in windows of different widths was studied due to the spatial variation of the thickness of the GaAs layers across the window in the SAE-L-N and SAE-L-W samples. Black curves in Figure 3 show the minimum and maximum of 12 measured GRE profiles across the window for each set of SAE-L-N and SAE-L-W samples grown under conditions corresponding to the St-L-L growth (18.8 nm/min). These profiles clearly show a pronounced GRE of up to 5–6 for narrower SAE-L-N samples, while a weaker GRE effect of about 2 is observed for wider SAE-L-W samples. In both samples, this effect decreases towards the window center. The red curves in Figure 3 show the results of GRE simulation for the SAE-L-N and SAE-L-W samples, which are in good agreement with the minimum experimental GRE profiles for both samples.

It should be noted that there are not any deposits over mask area, which means there are appropriate MOCVD growth conditions with low growth pressure and temperature, as well as high gas velocity, excluding the formation of elemental group III species on the dielectric mask [23,40,41]. In accordance with these background works, the GRE effect is attributed to lateral diffusion of material particles through the gas phase within the boundary layer, which is the limiting factor for the growth of semiconductors films with a typical migration length of adatoms on the surface of the order of only 1 µm [41]. The metalorganic particles can reach both the window and mask regions in the SAE samples. In the former, the particles are subjected to a pyrolysis reaction and participate in the growth of the epitaxial layer. In the latter, the particles may either be adsorbed onto the surface of the mask and migrate to the window region due to surface diffusion, or, for a short time, be desorbed from the surface. Desorbed particles return back into the gas phase and diffuse toward the window due to the negative gradient of gas concentration over the mask and window regions. As a result, the GRE increases in the narrower window due to the weaker spatial depletion of metalorganic group III flow along the width of this window, while this effect is reduced in the wider windows.

The observed 5-fold increase for GRE effect in the SAE-L-N means an extremely short duration (~3 s) of the SAE of QWs with a typical thickness ~6.5 nm in the InGaAs/GaAs heterostructures on the patterned substrates with a narrow width of window. This reduction of QW growth time will lead to an increase in the contribution of transient processes during the switching of the gas flows in the MOCVD reactor and the growth of graded index layers with an uncontrolled composition at the QW boundaries, which will worsen its radiative characteristics. Therefore, the analysis of the surface morphology and PL spectra for the samples grown with a narrow window was excluded from further study.

### 3.2. Surface Morphology of GaAs Layers

**Figure 4:**
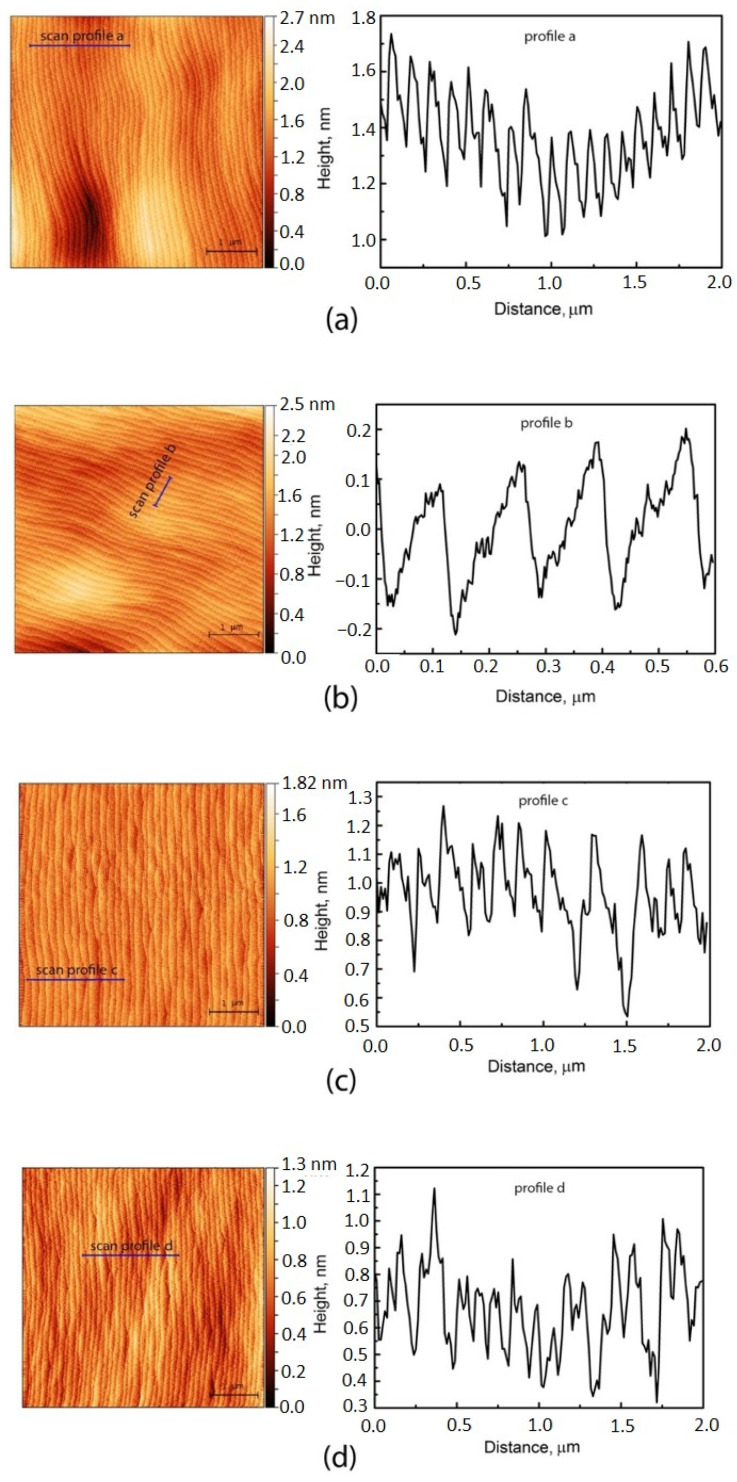

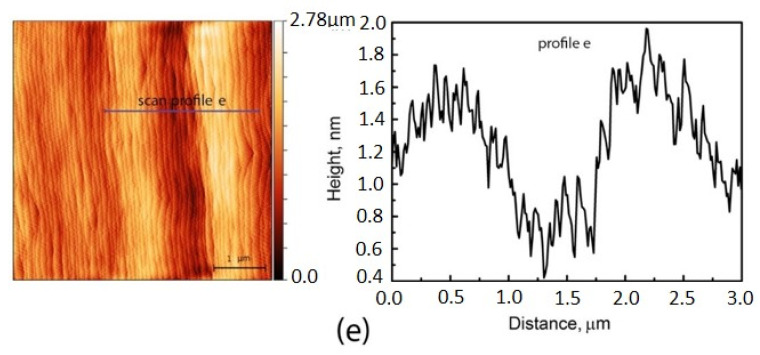
AFM images of GaAs layer surface (left) and scan profiles (right) for St-L-L (**a**), St-L-H (**b**) samples, and the SAE-L-W sample (**c**–**e**). The latter images (**c**–**e**) were obtained for A, B, and C points inside the window, as shown in Figure 1b.

To study the surface morphology, St-L-L and St-L-H samples were used, each with a thickness of 0.18 µm, which corresponds to the average thickness of the SAE-L-W sample measured with a profilometer. This was done in order to exclude the influence of the layer thickness on the surface morphology. Figure 4 shows 5 × 5 µm images of the GaAs layer surface and AFM scan profiles for St-L-L (a), St-L-H (b) samples, and SAE-L-W sample at 3 points (c–e) indicated in Figure 1b.

Figure 4a demonstrates a St-L-L surface with a monoatomic step ordering, having an average step height of 0.42 nm (1.5 ML) and regular terraces 120 nm wide, which correspond to the calculated width for the GaAs growth on a vicinal substrate with a misorientation angle of 0.2°. For the St-L-H sample (Figure 4b), the picture is similar: 0.28 nm (1 ML)-high steps, 100 nm-wide terraces, misorientation angle of 0.16°. In addition, these figures show the same step height and uniform step density over the surface of this layer. All these evidences confirm the realization of the step flow (SF) growth mechanism in this layer with a minimum step bunching, leading to an uneven distribution of the steps of different heights, including multiatomic ones. The influence of growth rate on growth mechanism has been described in [42]. Our results indicate that the growth mechanism does not change in the range of growth rates 18.8–37.6 nm/min. Most likely, the transition to a different growth mechanism under the growth parameters used occurs at higher growth rates.

However, step bunching (SB) becomes more pronounced for the GaAs layer grown inside wide windows of SAE-L-W sample. Figure 4c–e shows three regions with a different degree of SB, which can be distinguished in the AFM images. First, the lowest density of the steps is observed near the right window/mask interface (Figure 4c), while this density increases toward the window center with more regular distribution of the steps (Figure 4d). In the third region, located near the left window/mask interface, the structure with periodic sinusoidal changes of average surface height with a period and an amplitude of about 1.5 µm and 1.7 nm, respectively, is formed (Figure 4e). Although this surface also exhibits a fine ML-thick step structure, the highly irregular distribution of the narrow terraces indicates a strong step bunching in this region.

**Figure 5:**
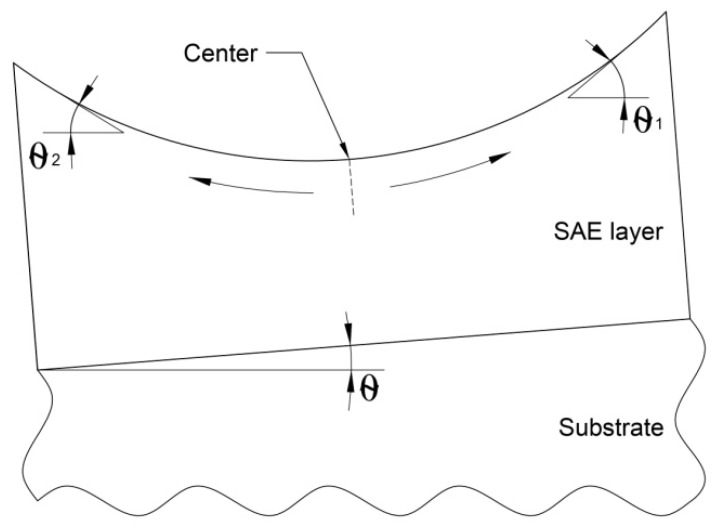
Schematic view of a SAE layer on a misoriented substrate.

As mentioned above, the growth rate can cause the transition from SF to SB. However, the sample St-L-H has been shown to have the same SF surface morphology as St-L-L. Taking GRE into account, the growth rate of the SAE-L-W sample corresponds to the growth rate of the St-L-H sample. Therefore, in our opinion, the main reason for the SB growth of the SAE-L-W sample is the misorientation of the layer because of the inhomogeneity of its thickness across the window caused by GRE. Figure 5 shows a schematic view of a layer grown by SAE on a misoriented substrate with a misorientation angle θ. It is seen that the layer has the same misorientation angle as the substrate only at one point, namely at the window center. The first angle of local misorientation of the layer (θ_1_) increases with respect to θ with a shift from the center towards the misorientation of the substrate (to the right). When shifting from the window center in the direction opposite to the direction of substrate misorientation (to the left), the second angle of local misorientation of the layer (θ_2_) first decreases and then changes sign and increases. In this case, the layer misorientation changes continuously. The maximum misorientation can be at the edges of the window. Thus, the SB enhancement near the mask/window interfaces in the SAE-L-W sample can probably be due to the variation of the GaAs layer thickness in these regions. Moreover, only an additional misorientation factor can explain a non-symmetrical effect for the surface morphologies observed near the opposite mask/windows interfaces in the SAE-L-W. It is well-known that increase of misorientation angle leads to SB enhancement, while an ideal SF growth mode is observed in the relatively narrow range of small misorientation angles (< 0.2°) [43]. Moreover, Peluchi et al. [44] and Moret et al. [45] demonstrated that increasing misorientation leads to transition from the SF growth mode with a ML-step and wide regular terraces at zeroth misorientation to numerous types of morphologies with various manifestations of the SB growth mode. Ultimately, the GaAs layer grown on the substrate with a maximum misorientation of 0.6° exhibits periodic SB profile (i.e., periodic sequence of differently step-bunched regions), as near the left window/mask interface in the SAE-L-W sample (Figure 4e).

### 3.3. Microphotoluminescence Spectra

**Figure 6:**
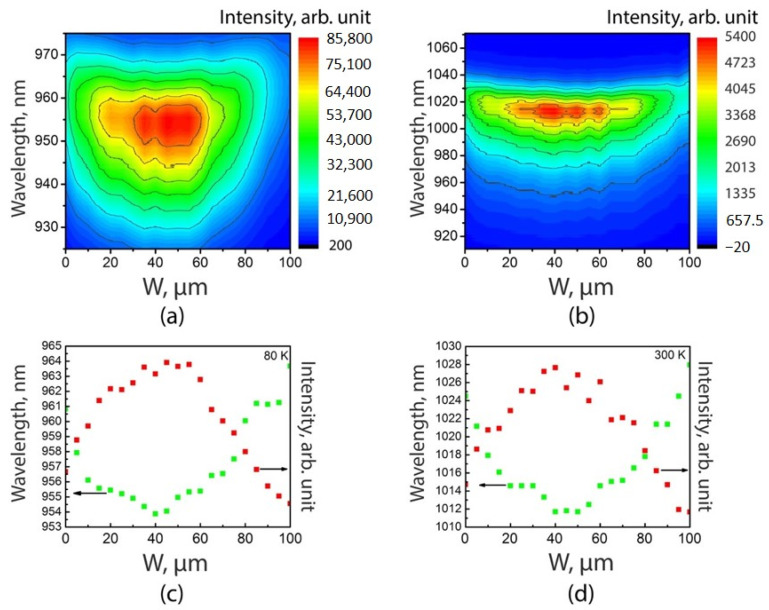
µ-PL spectra maps measured at 80 K (**a**) and 300 K (**b**) that show the spatial distribution of spectral position and intensity of the PL spectra of the InGaAs QW of the SAE-ML-W sample. PL spectra peaks and the corresponding wavelengths as functions of the position in the window obtained from µ-PL-maps measured at 80 K (**c**) and 300 K (**d**) for the SAE-ML-W sample. Green dot—wavelength, red dot—intensity.

Figure 6a,b show the maps of the µ-PL spectra for the SAE-*ML*-W sample at 80 K and 300 K, respectively. Using these maps, the maximum intensities and wavelength of µ-PL emission at 80 K and 300 K as functions of the position in the window are shown in Figure 6c,d, respectively. These maps and graphs demonstrate that the highest PL intensity is observed near the center of the 100-µm-width window and the spectral position of a PL peak exhibits a blueshift by 7–16 nm relative to the PL peak position at the edges of the window.

The observed variation in the positions of PL peaks can be explained by non-uniform distribution of thickness QW and its indium concentration across the width of window. Indeed, the lower the GRE in the center of the window, the thinner the QW grows. In addition, a lower effective diffusion length (D/k) of In compared to that of Ga leads to a higher mole fraction of In in the InGaAs QW at the window edge compared to the central region. Thus, both factors caused by non-uniformities in the QW thickness and indium concentration, blueshift the PL peak for spectra measured at the center of the window. These results agree with [33,46].

Decrease of PL intensities at the window edge can be attributed to the degradation of the GaAs surface morphology in these regions where both GRE and additional misorientation lead to enhancement of the SB (see Figure 4c,e), while this effect is less pronounced in the central part of the window (Figure 4d). The growth of an InGaAs/GaAs QW on a rough barrier layer leads to a deterioration in its optical quality. As a result, both a decrease in the PL intensity and a spectra broadening are observed at the window periphery regions. A slight asymmetry in the PL intensity distribution on the µ-PL map (Figure 6a,b) with a maximum intensity at a distance of 40 µm from the left mask/window interface corresponds to a better surface morphology at this interface as compared to the right one due to the various local misorientation effects inside the window (Figure 4c,e), described for Figure 5.

**Figure 7:**
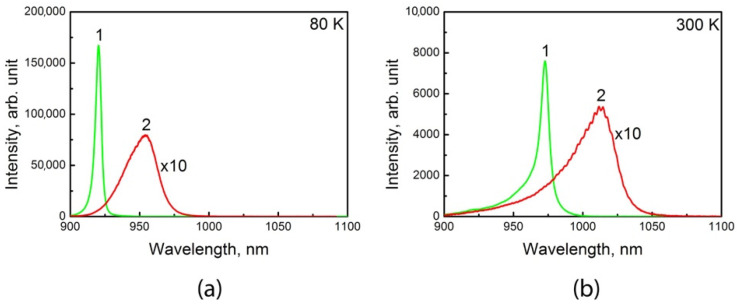
µ-PL spectra measured at 80 K (**a**) and 300 K (**b**) for St-ML (1) and SAE-ML-W (2) samples with InGaAs/GaAs QWs. For the SAE-ML-W sample, the spectra were taken at the window center.

In addition, we compared the maximum intensity PL spectra obtained in the central parts of the windows for the SAE-*ML*-W sample with the spectra measured for the St-*ML* sample, shown in Figure 7. The redshift of the SAE-*ML*-W sample spectrum relative to the St-*ML* sample spectrum can be explained by the fact that, at the same growth time and the same reagent fluxes, the QW of the SAE-*ML*-W sample is thicker than that of the St-*ML* sample due to GRE. The broadening and much lower intensity of the SAE-*ML*-W sample spectrum confirms the worse quality of the QW in this sample compared to the QW grown on the non-patterned substrate. Generally, these results correspond to a common concept of negative impact of the GRE and additional local misorientation on the surface morphology of a SAE GaAs layer and the optical properties of nanostructures with QWs obtained with SAE.

**Figure 8:**
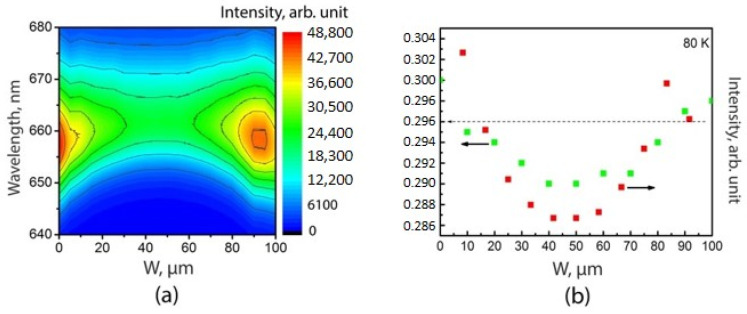
(**a**) µ-PL spectra map measured at 80 K for Al_x_Ga_1-x_As upper cladding layer for the SAE-ML-W sample. (**b**) Al mole fraction and µ-PL spectra intensity as functions of the measurement position in the window obtained from the PL spectra map. The dashed line corresponds to the Al mole fraction for the AlGaAs layer of StE sample. Green dot—composition, red dot—intensity.

Finally, we studied spatial distribution of PL emission from the AlGaAs upper cladding layer for the SAE-ML-W sample. Figure 8a shows the µ-PL (80 K) map for this layer, which was also used to determine the composition of the Al_x_Ga_1-x_As layer using the equation from [47]:

*E_Γ_*(*x*) = 1.519 + 1.155*x* + 0.37*x*^2^ − 5.41·10^−4^·*T*^2^/(*T* + 204), (12)

where *E_Γ_* is the AlGaAs bandgap; *x* is the Al mole fraction; *T* is the temperature.

Figure 8b shows distributions of both the Al mole fraction and the PL intensity across the window. It should be noted that there is a very slight change (1 mol.%) of Al mole fraction over the window and its value is practically equal to the Al-content in the standard MOCVD epitaxy without a mask (StE) sample. The results demonstrate the growth of compositionally homogeneous AlGaAs epitaxial layers in ultra-wide windows.

The homogeneity of a bulk layer composition is an important factor that affects the characteristics of the heterostructure and devices based on them. For example, if we consider semiconductor lasers, then the bulk layers of the waveguide and cladding layers forming the waveguide for the laser mode can usually have an Al mole fraction difference of the order of 10–30% [48]. In the case when the AlGaAs layer performs the function of a cladding, the determined difference of 1% will not make a noticeable contribution to the change in the laser mode shape.

## 4. Conclusions

It is shown that an improvement of radiation characteristics of a QW structure obtained using the SAE in the ultra-wide windows requires optimization of the mask pattern parameters, including the narrower width of mask, to ensure minimal variation of the GRE between the window center and the window edge. In addition, in order to reduce the layer misorientation, it is necessary to optimize the thickness of the lower part of the waveguide part grown using the SAE.

This study showed that the GaAs layers grown using the SAE have a morphology different from that of the StE GaAs layers at identical MOCVD growth conditions. The surfaces of the layers grown using SAE and StE layers correspond to the SB and SF growth modes, respectively. The PL spectra obtained for the QW grown by SAE have an order of magnitude lower intensity and a much larger half-width compared to the PL spectra of QW grown by standard epitaxy. In our opinion, one of the reasons for this is the change in the type of surface morphology from SF to SB at the growth of QW structures using StE and SAE, respectively.

The results obtained suggest the possibility of using SAE in the case of ultra-wide windows to create different types of light-emitting devices. On the one hand, if we are talking about lasers with a fixed line of optical radiation, then the observed tuning of the PL wavelength of the QW (Figure 6) is a negative effect. In this case, optimization of the mask and window widths is still required to reduce the difference for GRE in the outermost and center regions of the ultra-wide window. On the other hand, the effect of the luminescence wavelength variation across the window is useful in various types of tunable laser sources; in particular, in high-power tunable semiconductor lasers with a wide aperture [49,50]. A 100-nm-wide tuning was demonstrated in [50] for a high-power semiconductor laser with a 100-µm-wide aperture based on a planar QW with a constant composition and thickness. The tuning range was limited by the width of the gain spectrum of the planar QW. The results obtained in this work show that SAE QW exhibit a change in the PL wavelength across the window. This indicates a change in the composition and thickness of the QW over the window, and, as a consequence, a wider spectrum of material gain. This effect can be used to expand the tuning range of laser sources. The ability of a smooth change in the wavelength across the ultra-wide window region is also important for multi-wavelength laser arrays of single-mode laser emitters required for dense wavelength division multiplexing (DWDM) to increase data transfer rate [51,52].

## Figures and Tables

**Table 1:** Description of experimental samples.

Sample	Epitaxy Type	Composition	Window/Mask µm	Growth Rate GaAs, nm/min	Growth Time
St-L-L	Standard	GaAs layer	-	18.8	5 min 20 s
St-L-H	Standard	GaAs layer	-	37.6	10 min 40 s
St-ML	Standard	AlGaAs/GaAs/InGaAs multilayer structure	-	18.8	10 s ^2^
SAE-L-N	Selective	GaAs layer	60/340	90.2–99.6 ^1^	2 min
SAE-L-W	Selective	GaAs layer	100/100	32.9–37.4 ^1^	4 min
SAE-ML-W	Selective	AlGaAs/GaAs/InGaAs multilayer structure	100/100	32.9–37.4 ^1^	10 s ^2^

^1^ GaAs growth rate taking into account the minimum and maximum growth rate enhancement (GRE) profiles in the center of the window. ^2^ QW growth time.

## Data Availability

The data presented in this study are available in article.
